# Gut Microbiota-Assisted Synthesis, Cellular Interactions and Synergistic Perspectives of Equol as a Potent Anticancer Isoflavone

**DOI:** 10.3390/ph15111418

**Published:** 2022-11-16

**Authors:** Hardeep Singh Tuli, Ajay Kumar, Katrin Sak, Diwakar Aggarwal, Dhruv Sanjay Gupta, Ginpreet Kaur, Kanupriya Vashishth, Kuldeep Dhama, Jagjit Kaur, Adesh K. Saini, Mehmet Varol, Esra Capanoglu, Shafiul Haque

**Affiliations:** 1 Department of Biotechnology, Maharishi Markandeshwar Engineering College, Maharishi Markandeshwar (Deemed to be University), Ambala 133207, India; 2 Punjab Biotechnology Incubator (PBTI), Phase VIII, Mohali 160071, India; 3 NGO Praeventio, 50407 Tartu, Estonia; 4 Department of Pharmacology, Shobhaben Pratapbhai Patel School of Pharmacy and Technology Management, SVKM’s, NMIMS, Mumbai 400056, India; 5 Advance Cardiac Centre Department of Cardiology, Post Graduate Institute of Medical Education and Research (PGIMER) Chandigarh 160012, India; 6 Division of Pathology, ICAR-Indian Veterinary Research Institute, Izatnagar 243122, India; 7 Graduate School of Biomedical Engineering, ARC Centre of Excellence in Nanoscale Biophotonics (CNBP), Faculty of Engineering, The University of New South Wales, Sydney 2052, Australia; 8 Department of Molecular Biology and Genetics, Faculty of Science, Kotekli Campus, Mugla Sitki Kocman University, Mugla 48000, Turkey; 9 Department of Food Engineering, Faculty of Chemical and Metallurgical Engineering, Istanbul Technical University, Istanbul 34469, Turkey; 10 Research and Scientific Studies Unit, College of Nursing and Allied Health Sciences, Jazan University, Jazan 45142, Saudi Arabia

**Keywords:** cancer, natural compounds, gut microbial synthesis, plant flavonoids, equol

## Abstract

It is well known that, historically, plants have been an important resource of anticancer agents, providing several clinically approved drugs. Numerous preclinical studies have shown a strong anticancer potential of structurally different phytochemicals, including polyphenolic constituents of plants, flavonoids. In this review article, suppressing effects of equol in different carcinogenesis models are unraveled, highlighting the mechanisms involved in these anticancer activities. Among flavonoids, daidzein is a well-known isoflavone occurring in soybeans and soy products. In a certain part of population, this soy isoflavone is decomposed to equol under the action of gut microflora. Somewhat surprisingly, this degradation product has been shown to be more bioactive than its precursor daidzein, revealing a strong and multifaceted anticancer potential. In this way, it is important to bear in mind that the metabolic conversion of plant flavonoids might lead to products that are even more efficient than the parent compounds themselves, definitely deserving further studies.

## 1. Introduction

During the last decade, the role of gut microbiota in the metabolism of plant food-derived flavonoids has been highlighted [1]. While the structural characteristics and biological effects of many catabolites are still unknown, one of the best-described examples revealing the formation of more active agents within colonic degradation is the conversion of soy isoflavone, daidzein (4′,7-dihydroxyisoflavone). In fact, daidzein is metabolized to equol (4′,7-isoflavandiol) under the action of intestinal microflora; however, the knowledge about the exact bacterial species involved in this process is still limited [2]. Moreover, only about 30% of the Western population and 60% of the Asian population have been shown to be able to produce equol [3], whereas only the *S*-enantiomeric form of equol is generated within the colonic degradation of daidzein in the human gut [4]. 

A range of in vitro and in vivo studies have demonstrated numerous important bioactivities of equol in the model systems of several common diseases, including different types of malignancies. Recent studies have indeed shown strong anticancer effects of equol, inhibiting the development of both hormone-dependent malignancies such as breast and prostate tumors [5,6], as well as hormone-independent neoplasms such as gastric cancer [7], hepatocellular carcinoma [8] and non-small cell lung cancer [9]. Different molecular mechanisms behind these effects have been described, including the suppression of proliferation, migration and invasion of malignant cells besides promoting also their apoptotic death, through attacking diverse molecular targets and regulating a variety of cellular signal transduction pathways [6,7,10,11,12]. Several studies have also demonstrated the ability of equol to modulate the hormonal balance by binding to estrogen receptors (ERs), doing it with a higher affinity than its parent compound daidzein [2].

In this review article, the chemistry of equol and its biosynthesis from daidzein are described, discussing also the important biological activities of equol in different types of malignant tissues. Additionally, the synergistic effects of equol with clinically used standard anticancer drugs are under discussion to reveal the possibilities for a reduction in adverse side effects by lowering the doses of chemotherapeutics by co-administering with equol. Finally, the advances of nanotechnology in improving the delivery of equol directly to the malignant sites are presented, opening additional possibilities for decreasing the drug-related toxicities to normal healthy tissues. It is expected that all these efforts together might bring us a step closer to the development of more efficient cancer therapies in the future. 

## 2. Biosynthesis, Absorption to Metabolism

Equol, a potent isoflavone, has steadily been gaining pharmacological importance, owing to the wide range of benefits that it offers. Bacterial daidzein conversion in the intestine yields *S*-equol, which has been shown to exhibit greater stability, absorbance and a lowered rate of clearance, as compared to its parent drug [2]. This enantiomeric form of the compound has been linked with promising activity on sex-hormone receptors, and the modulatory effects exerted have been related to the amelioration of a variety of metabolic disorders, such as cancer, cardiovascular disease and neurodegenerative conditions [13]. Of all the known isoflavones in nature and their metabolites (Table 1 and Table 2), equol has been observed to have the highest affinity for estrogen receptors [14]. 

**Figure 1:**
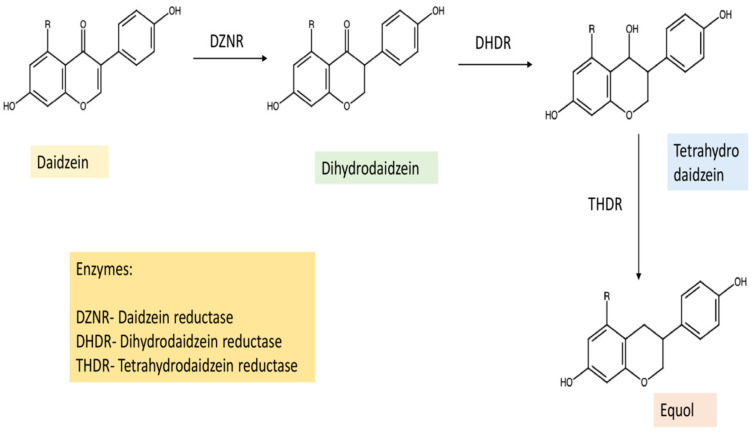
Diagrammatic representation of equol biosynthesis with the enzymes involved.

An increased focus on research has led to the discovery of a number of bacterial strains capable of equol biosynthesis, primarily concentrated in the intestinal microbiota. Most of these microorganisms are anaerobic in nature, belonging to the family *Eggerthellaceae*. Dihydrodaidzein and tetrahydrodaidzein are produced as intermediates in the process of synthesis, and the reactions are mediated by reductase enzymes, primarily occurring in oxygen-susceptible bacteria [49]. These include daidzein reductase, dihydrodaidzein reductase, tetrahydrodaidzein reductase and dihydrodaidzein reductase (linked with an increase in equol biosynthesis). In addition to this, related organisms belonging to the class *Coriobacteria* have been linked with equol production as well, and four key enzymes have been identified, as aforementioned [50]. The biosynthetic pathway may be summarized as shown in Figure 1.

A study conducted by [51] evaluated the effect of the administration of HXMB408 bacteria solution on daidzein metabolism, and the subsequent equol production, using a rat model. The results from the experiment indicated an increase in equol production in the test group as compared to the control group (both administered with daidzein), signifying a degradative role of bacteria on isoflavones. An improved microbiome environment, which is microbially diverse, has been linked with improved metabolite production [51].

An interesting trial conducted by [52] aimed to establish a relationship between the equol production in children and its link with maternal equol production, and it was observed that a child’s equol production status (producer/non-producer) bears a direct link with the mother’s status, as opposed to other factors such as dietary intake or excreted levels of the metabolite [52]. 

In a study undertaken by [53], the metabolism of daidzein was explored using an in silico and physiologic based model. The fecal samples used to mimic the gut microbiome as well as the rat model employed indicated the potent estrogenic nature of *S*-equol; however, lower plasma concentrations than the parent isoflavone were observed. This may vary on the basis of soy supplementation as well as isoflavone content in different types of diet, indicating the relation between dietary supplementation and equol production [53]. In a separate comparative study between equol producers and non-producers, it was observed that an increase in daidzein intake was linked with an increased level of *Assaccharobacter celatus* and *Slackia isoflavoniconvertens*, which are gut microbes associated with equol production [54], further supplementing the influence of dietary intake. 

As we continue to discover the intestinal microbes responsible for equol production, it is important to note that inter-individual differences account for variation in the composition of the intestinal microbiota [55]. Owing to this, only about one-third of the world’s population is capable of equol biosynthesis, by the metabolism of daidzein [56] Advancements in biotechnological research would help in designing technological strategies to enhance the output and provide a sustainable source for equol to keep pace with its growing therapeutic benefits in biological systems.

## 3. Structure-Activity Relationship of Equol

In addition to being distinguished on the basis of a 3-phenylchromone structure, isoflavones are further classified as aglycones and glycosides—daidzein belonging to the former category. The affinity of isoflavones to estrogen receptors may be attributed to their structural similarity to estrogen [57]. A structure activity relationship (SAR) study conducted by Cho et al. [58] revealed that an increased saturation of the C-ring in the structure of isoflavones enhanced their ERβ selectivity, enhancing transactivation and exerting a neuroprotective effect. Among the metabolites of daidzein, equol and dehydroequol were observed to exert the most potent neuroprotective effect, and it was revealed that minor modifications to the C-ring could help to alter the activity of these compounds, which may be used in the synthesis of anti-inflammatory analogues for the management of a variety of metabolic conditions [58]. Equol is an optically active molecule, owing to its non-planar structure, and the two phenolic groups present in its structure may be potential sites for tyrosinase activity, yielding quinones. These oxidation products have been observed to exert pro-oxidant activity [59]. 

## 4. Equol Production at a Laboratory and Industrial Scale

The industrial scaling-up of equol production has been limited due to the oxygen susceptibility and fastidiousness of the biosynthetic bacteria [60]. However, researchers have attempted to overcome this drawback by carrying out the cloning of equol-producing genes in certain microbes and expressed them in host organisms. A study by Vázquez et al. [61] explored the genes coding for major enzymes involved in equol production, in *Adlercreutzia equolifaciens* DSM19450T. This was followed by the cloning of the genes and expression in *Escherichia coli* hosts, offering an avenue for biotechnological advancement [61]. In addition to this, *Lactobacillus intestinalis* has been observed to effectively yield equol from daidzein, as it expresses the genes of the enzymes required in its production [62].

As previously discussed, the main barriers to scaling-up and the industrial production of equol include bacterial constraints; however, advances have led to the exploration of alternate strategies for equol production. In a study conducted by Kawada et al. [63], it was observed that a cluster of three genes is associated with the conversion of daidzein to *S*-equol in *Eggerthella* sp. YY7918, a bacterium found in human feces. It was observed that the gene sequence responsible for production was similar to that of *Lactococcus*, indicating the potential to explore other species for the industrial production of equol. A study conducted by Mustafa et al. [64] highlighted the effects of various physiological conditions such as pH, temperature, and inulin concentration on the production of isoflavones in soymilk by *Bifidiobacterium* sp.; temperature was found to be a key factor influencing daidzein production. This may be indicative of the fact that the optimization of temperature conditions in industrial settings may enhance isoflavone production. Drawbacks of traditional production techniques include harnessing non-productive strains found intestinally; however, the development of recombinant strains has paved the way for the effective aerobic production of *S*-equol. Although a major drawback included a limited yield owing to limited isoflavone solubility, a study undertaken by Lee et al. [65] explored the utilization of hydrophilic polymers and polar aprotic solvents to considerably improve the solubility of these compounds. This indicates that the inclusion of these compounds may enhance the production of these compounds and improve the outcomes on an industrial scale.

## 5. Equol as a Potent Anticancer Agent

### 5.1. Apoptotic and Cell Cycle Arrest Mechanisms

**Figure 2:**
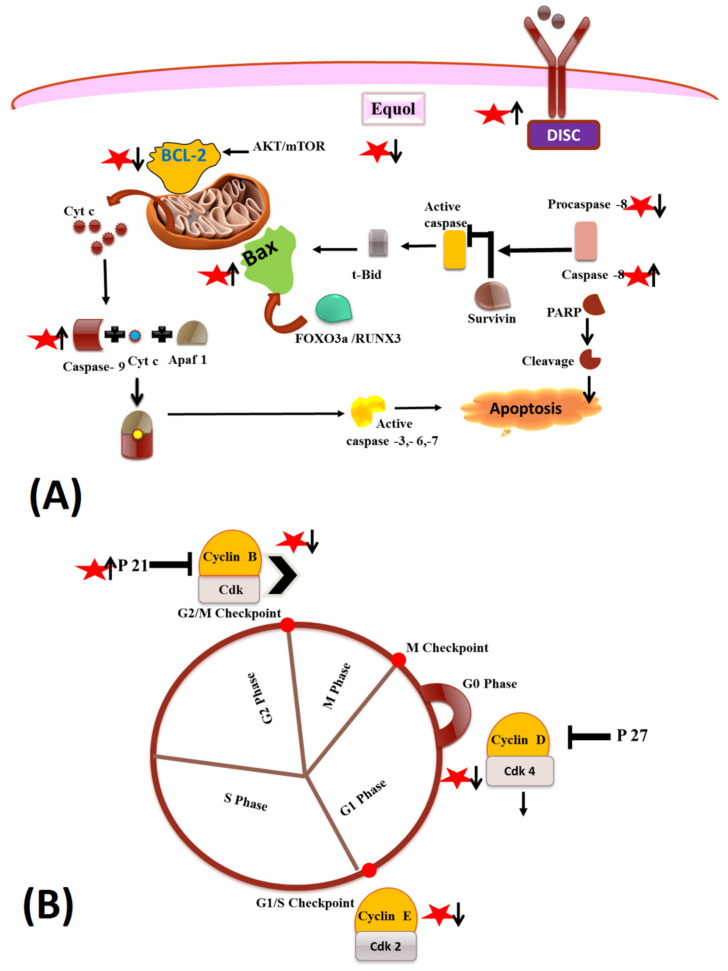
(**A**) The effect of equol (represented as red star) on apoptosis. (**B**) The effect of equol on regulation of cell cycle progression.

Equol is being consumed and marketed as a dietary nutraceutical agent with promising anticancer activities [66,67,68]. Numerous studies have highlighted the protective role of equol in different hormone-dependent and -independent cancer cells, such as breast, gastric, prostate cancers, etc. Studies have attributed the anti-cancerous properties of equol via its apoptotic and cell cycle arrest mechanisms. Apoptosis, being a fundamental process, is essential for normal development and in the maintenance of tissue homeostasis [69,70]. Studies have shown that targeting different signaling intermediates in apoptosis-inducing pathways may prove beneficial in cancer prevention and therapy [71]. Different studies have depicted the apoptotic and anti-proliferative properties of equol alone or in combination with available anticancer drugs/herbal formulations in in vitro and in vivo models of cancer [71,72]. Equol has been shown to induce apoptosis via both extrinsic and intrinsic pathways of apoptosis involving different biological mechanisms in divergent cancer models; further equol was shown to arrest cell cycle progression via the involvement of different cell cycle check points (Figure 2). The phytohormone property of equol has proved to be beneficial in many in vitro breast cancer studies as it is reported to bind to both estrogen receptors i.e., ERα and ERß, and is implicated in the inhibition of proliferation and induction of apoptosis in breast cancer cells. Further studies have also reported that equol induces apoptosis in ER-negative breast cancer cells [69,71,72,73]. In a study, [23] delved deep into the cytotoxic effect of equol in MCF-7 breast cancer cells, delineating the apoptotic pathways and proteins involved. The study depicted the involvement of cleaved caspases-9,7 and the subsequent release of cytochrome C into the cytosol and its subsequent action on cytosolic targets, thereby inducing apoptosis. Moreover, the combined effect of equol and tamoxifen induced a time-dependent reduction in bcl-2 expression, thereby the bcl-2: bax ratio was reduced by the combination of the two compounds. These findings are consistent with previous studies on breast cancer cell lines. Various studies have also reported the role of the equol-mediated intrinsic pathway of apoptosis in different in vivo and in vitro cancer models [23,74]. For instance, in vitro studies have depicted the inhibitory role of equol in a time- and dose-dependent manner to arrest cell cycles [75]. On human gastric carcinoma cells MGC-803, the role of equol was elucidated in inhibiting cell cycle proliferation at the G0/G1 phase by regulating CDK2/4, Cyclin D1/E1, and P21 expression. Further, equol induced apoptosis in the cells via the cleavage of PARP and caspase-3. The study also depicted the role of AKT-mediated cell cycle arrest and apoptosis. Several studies have shown the effect of different isoflavones and equol in regulating cell cycles via reducing the activity of the Cyclin B/CDK complex, thereby inhibiting cell proliferation. In different prostate cancer in vitro studies, equol was associated with the activation of FOXO3a, one of the forkhead-family factors of transcription involved in apoptosis via the protein kinase B (Akt)-specific signaling pathway [76]. The studies available on the role of equol in inducing apoptosis and cell cycle arrest on different types of cancer cells are promising but limited; there is a requirement of more delineated and elucidated investigations involving commonly occurring cancers and at different doses of equol alone or in combination with the commonly available chemotherapeutic agents are needed along with the approach of translational research from bench side to bed side [77,78,79,80].

### 5.2. Antiestrogenic Action

**Figure 3:**
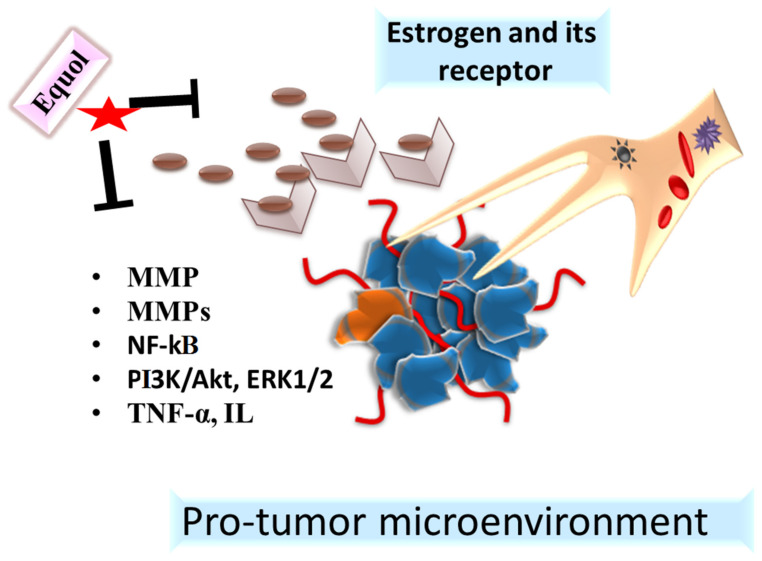
Anticancer action of equol through modulating its binding to estrogen receptors. Red 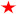 star indicated the Equol.

Estrogens are the hormones that play an important role in the development of the female reproductive system as well as secondary sex characters. However, when there is an excessive production of estrogens in the body, it may lead to problems such as fibroids, weight gain, and breast cancer. The excessive estrogen levels are known to cause interference with the DNA, leading to the development of cancerous cells [81,82]. Unbalanced production of estrogens may lead to the formation of catechol estrogens (4-hydroxyestrone, 4-hydroxyestradiol, 2-hydroxyestrone, and 2-hydroxyestradiol) and 16α-hydroxylation to 16α-hydroxyestrone [81,83]. The oxidation of 4-hydroxyestrone and 4-hydroxyestradiol to estrone-3,4-quinone and estradiol-3,4-quinone leads to the formation of depurinating adducts by interacting with the host DNA, which in turn leads to the generation of apurinic sites. These apurinic sites then lead to the error-prone base excision repair-causing mutations in the DNA, resulting in the onset of prostate and breast cancers [83]. However, anti-estrogenic metabolites such as equol have been known to reduce the risk of development of cancer by metabolizing the estrogens and facilitating their excretion through urine [84,85]. Equol is an isoflavone metabolite that is produced by the gut bacteria as a result of soy protein digestion [86,87]. However, the mechanism how its production differs in individuals is not understood completely [88]. It exhibits its anti-estrogenic activity by binding to the α and β estrogen receptors [84] and is known to provide relief from menopausal symptoms such as hot flashes [88,89]. In addition, the binding of equol with the ER receptor has been linked to the reduction in breast cancer cell growth and the activation of apoptosis. Lathrop et al., 2020, reported S-equol as a well-tolerated oral ER-Beta agonist in patients with triple negative breast cancer (TNBC), and inhibited proliferation via the downregulation of nucleoprotein Ki-67 [90]. The endogenously produced equol differs from the exogenously produced one as the gut bacteria produce *S*-equol, whereas the synthetically produced equol is the racemic *R*/*S*-(±) mixture [91]. The enantiomers of equol are distinguished based on their binding mechanism to estrogen receptors, where *S*-equol binds to α estrogen receptors and *R*-equol binds to β estrogen receptors [92,93]. In a study, the effect of *R*/*S*-(±) equol, *S*-equol, and soy isoflavone was explored on the reproductive tract of adult apoE-null mice population. It was found that an anti-estrogenic effect was observed in mice administrated with both *R*/*S*-(±) equol and *S*-equol, irrespective of the soy isoflavone dosage [88]. It was concluded from this work that the equol production in the mice was dependent on the gut bacteria rather than the soy isoflavone-dependent diet. However, in humans, the anti-estrogenic effect of equol was dependent on the soy consumed in the diet [94,95,96]. Thus, equol can be used for reducing the risk of development of various cancers such as breast cancer due to its anti-estrogenic properties (Figure 3). 

### 5.3. Anti-Angiogenic and Anti-Metastasis Activities

**Figure 4:**
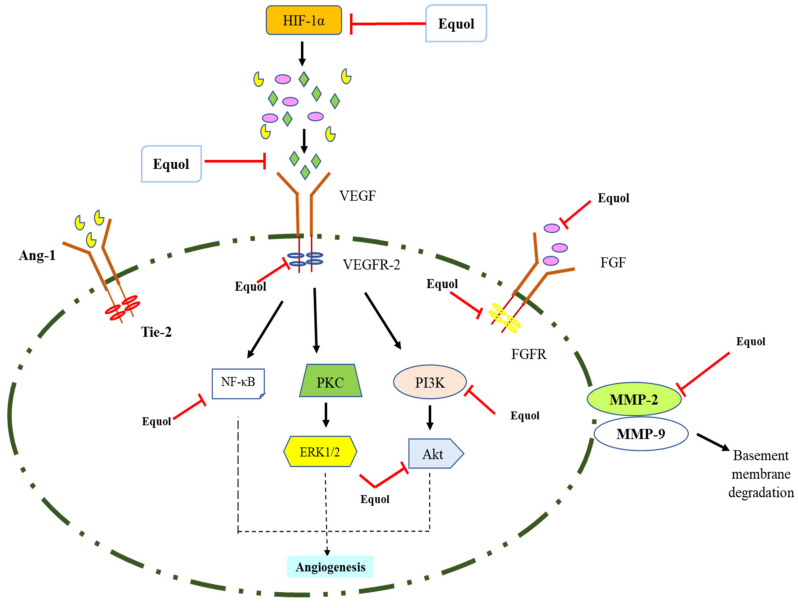
Major signaling pathways targeted by equol in angiogenesis and metastasis processes.

Cancer is one of the prime health issues in the world, requiring an effective strategy to battle its rising incidence and mortality rates [97]. In all forms of malignancy, there is a higher invasive potential and poor mortality relating to angiogenesis and metastasis [12]. The main characteristics of cancer are changes in vascular architecture and uncontrolled angiogenesis. Angiogenesis is the process of forming new blood vessels from existing ones, which is regulated by positive (angiogenic) and negative (anti-angiogenic) endogenous factors such as adult endothelial cells (ECs) [98]. Angiogenesis is linked to the development of numerous illnesses and plays an important part in normal physiological activities such as embryo development, wound healing, and the menstrual cycle [99]. The dysregulation of angiogenesis is widely established to cause the onset and progression of a variety of illnesses, including rheumatoid arthritis, psoriasis, diabetic retinopathy, malignant tumors, and age-related macular degeneration (AMD) [100]. Angiogenesis is essential for the beginning, growth, and spread of malignancies because tumor cells require oxygen, nutrition, and growth factors for proliferation and development [101]. Tumors maintain blood supply by generating chemical signals that cause angiogenesis to occur [102]. A vital equilibrium between numerous pro-angiogenic and anti-angiogenic factors is required for angiogenesis, and a shift in this balance can result in pro- or anti-angiogenic consequences [103]. Inflammation, ischemia, hypoxia, and other circumstances that act on cytokines, as well as angiogenic factors such as vascular endothelial growth factor (VEGF) or fibroblast growth factor (FGF) generated by tumors and other defective cells, can all trigger angiogenesis [104]. Pro-angiogenic factors such as vascular endothelial growth factor-A (VEGF-A), basic fibroblast growth factor (bFGF), thymidine phosphorylase (TP), metalloproteinase-9 (MMP-9), urokinase-type plasminogen activator (uPA), and adrenomedullin (ADM) have the main roles in angiogenesis (Figure 4) [105]. As a result, inhibiting angiogenesis appears to be a promising therapeutic strategy for the treatment of a variety of disorders, including cancer [106]. Although anti-angiogenic medications such as bevacizumab, pegaptanib, ranibizumab, sunitinib, sorafenib, regorafenib, and axitinib are available, they all have substantial side effects such as cardiovascular toxicity, bleeding risk, intraocular inflammation, ocular hemorrhage, and retinal detachment [107]. As a result, novel and effective medicines targeting angiogenesis with fewer adverse effects that complement and can be coupled with existing medications are needed to be developed/explored [108].

Researchers have focused on potent, naturally occurring anti-angiogenic molecules because these inexpensive constituents have minimal toxicity—they have been used as a natural remedy for centuries in different parts of the world for curing various disorders [109]. Among the broad spectrum of natural products, soybean is one of them and daidzein is one of the potent isoflavones present in it. Daidzein can be metabolized by specific flora in the gut to produce equol [110]. Equol is a bioactive metabolite of daidzein with higher biological activity than daidzein. Equol has numerous pharmacological activities such as anticancer, neuroprotective, and anti-angiogenesis [111]. Bellou et al. [112] reported that 6-methoxyequol (6-ME) reduced the growth of human endothelial cells from umbilical vein (HUVEC) by downregulating the expression of VEGF- and FGF2 through the phosphorylation of MEK1/2 and ERK1/2 via the mitogenic MAPK pathway. 6-ME significantly reduced neovascularization and tumor volume in BALB/c nude mice A-431 xenograft tumors, showing that its anti-angiogenic potential is useful in cancer chemoprevention. Ref. [113] investigated that equol-treated MGC-803 cells revealed a significant downregulation of Ki67, CDK2/4 and Cyclin D1/Cyclin E1, whereas the upregulation of P21WAF1, PARP, caspase-3, and P-Akt (Ser473 and Thr308) led to arresting cells at G_0_/G_1_ phase, showing its anticancer and apoptosis-inducing potential. Ref. [23] reported that the combination of equol and tamoxifen significantly inhibited the proliferation of MCF-7 cells and induced apoptosis in MCF-7 more efficiently than each compound alone. The combination treatment in MCF-7 cells significantly upregulated the levels of PARP, caspase-9, caspase-7, and α-fodrin cleavage, and downregulated the bcl-2: bax ratio, showing the apoptosis-inducing potential of equol and tamoxifen. Ref. [114] investigated that equol and daidzein significantly inhibited the migration and invasion of prostate cancer cell lines (DU-145 and PC-3) by downregulating the MMP-2 and MMP-9 expression. Therefore, these results suggest that equol could inhibit the pathway of angiogenesis via associated signaling markers and might serve as a therapeutically potent antitumor agent. 

### 5.4. Antioxidant and Anti-Inflammatory Effects of Equol

Equol has attracted the attention of many researchers due to its substantial antioxidant properties; its strong antioxidant effects have been proven in many experimental models [115]. Comparative studies with many compounds that are accepted to have antioxidant properties have shown that equol is a superior antioxidant and has a higher antioxidant capacity than antioxidant compounds such as vitamin C, vitamin E, quercetin, ascorbic acid, and genistein [116]. It has been determined that the macrophage-protective activity of equol against oxidative stress is achieved by decreasing the lipid peroxidation product malondialdehyde (MDA), increasing the activities of the antioxidant enzymes superoxide dismutase (SOD) and glutathione, and suppressing the effect of L-lactate dehydrogenase or oxLDL [117]. There are also many papers about equol showing antioxidant properties in other tissues and cells. For example, equol protects rats against focal cerebral ischemia by significantly reducing oxidative stress, and the other studies provided that equol causes an increase in SOD, catalase, acetylcholinesterase and glutathione peroxidase activities, and decreases MDA levels and superoxide production in mice [118,119,120]. In a paper examining the molecular mechanism of antioxidant activity of equol, it has been indicated that equol inhibits activator protein 1 (AP-1), a substantial molecule of the oxidative stress chain, and thus, exerts an anti-tumor effect by suppressing the cell transformation directed by the MEK signaling pathway [121,122]. Moreover, it has been reported that intracellular superoxide anion and hydrogen peroxide production is decreased by equol in phagocytic cells, toxic effects caused by increased ROS in neutrophils are eliminated, and the phosphorylation of NADPH oxidase regulatory proteins is decreased by equol [123,124]. Additionally, numerous studies offered that equol supports the expression of antioxidant genes, increases nuclear factor erythroid 2 (Nrf2) transcripts, increases SOD activities, shows protective activity against hydrogen peroxide-induced cell death, and prevents oxidative stress-induced apoptosis and vascular damage in epithelial and endothelial cells [125,126,127,128]. In primary cortical neuron cells, it was determined that equol decreased the amount of the reduced/oxidized glutathione [129]. 

**Figure 5:**
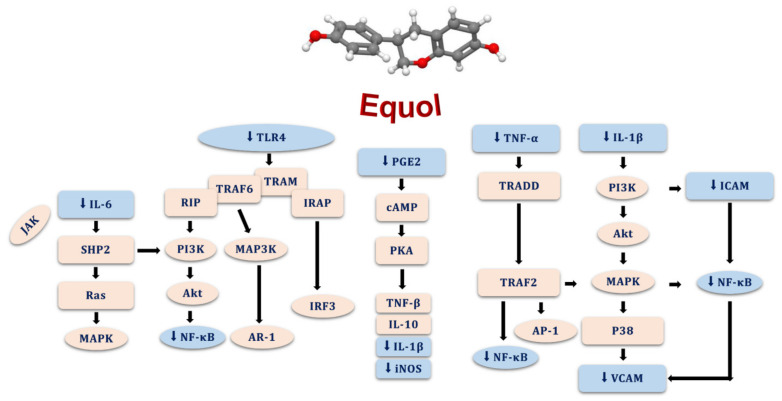
Anti-inflammatory activity mechanisms of equol. Akt: protein kinase B, AP: activator protein, AR: androgen receptor, cAMP: cyclic adenosine monophosphate, ICAM: intercellular adhesion molecule, IL: interleukin, IRAP: insulin-responsive aminopeptidase, IRF: IFN regulatory factor, iNOS: nitric oxide system, JAK: janus kinase, MAPK or P38: mitogen-activated protein kinase, NF-κB: nuclear factor kappa B, PGE2: prostaglandin E2, PI3K: phosphoinositide 3-kinase, PKA: protein kinase A, SHP: Src homology-2 domain-containing protein tyrosine phosphatase, TLR: Toll-like receptor, TNF: tumor necrosis factor, TRADD: TNFR1-associated death domain protein, TRAF: TNFR-associated factor, TRAM: TRIF-related adaptor molecule, VCAM: vascular cell adhesion molecule.

The data obtained as a result of numerous studies have shown that continued oxidative stress causes chronic inflammation, resulting in many diseases, including cancers [130,131,132]. Known as a pro-inflammatory factor expressed in all cell types, NF-κB is involved in the oxidative stress mechanism by regulating the expression of many genes playing substantial roles in the pathology of inflammatory diseases [133,134]. Equol has been identified as a NF-κB inhibitor (Figure 5) along with the down-regulation of the production of various kinds of cytokines (e.g., TNF-α, IL-1, IL-6 and IL-8) and inflammatory biomarkers (e.g., prostaglandin E2, COX-1 and MCP-1) in macrophages, and suppresses the increase in free radicals such as nitric oxide [135,136,137,138,139]. Additionally, it was determined that equol suppresses the release of IL-6 and TNF-α in microglia cells and mediates the inhibition of MAPK, NF-κB, and Toll-like receptor 4 [140,141]. It has been also reported that equol decreases NO synthase expression and nitric oxide production in astrocytes [142]. On the other hand, there are published papers about the anti-inflammatory activities of the isomers of equol named racemic equol, *S*-equol, and *R*-equol; the studies indicate that *S*-equol is less potent on inflammation than racemic and *R*-equol, of which inhibit the inflammatory factors such as IL-1, IL-6 and COX-1 expressions [135,143]. Consequently, equol appears to have significant potential as an antioxidant and anti-inflammatory natural isoflavandiol estrogen, and more studies are clearly needed to gain a detailed understanding of the molecular targets and molecular activity mechanisms of equol and its isomers in oxidative stress formations and inflammatory processes.

## 6. Synergism with Anti-Cancer Agents

The effectiveness of therapeutic anticancer treatments is currently hampered by the prevalent incidence of multidrug resistance. Combinatorial therapy has, therefore, received a lot of interest in the field of cancer therapy, with researchers trying to understand how a combination of anticancer drugs might behave either additively or synergistically to offer increased antitumor activity at lower doses than single-drug therapy. Recent advances in medical research have made synergy assessments a crucial area for improving therapeutic efficacy and influencing multiple targets in addition to just one. Equol has shown promising anticancer capabilities, and various studies have been carried out to assess its potential to be combined with other chemotherapeutic drugs to provide synergistic effects. 

Kim and Kim demonstrated that treating HeLa cells with TRAIL (Tumor necrosis factor-related apoptosis-inducing ligand) and equol made them more susceptible to TRAIL-mediated apoptosis. Caspases-3, -8, and -9 activation and BID cleavage by equol increased TRAIL-induced apoptosis. Additionally, DR4/Fc chimaera protein and DR5/Fc chimaera protein effectively decreased the caspases’ activation, BID cleavage, and apoptotic cell death brought on by equol and TRAIL co-treatment [144]. 

A study by Charalambous et al. [23] reported that 4-hydroxy-tamoxifen (4-OHT; >100 nM) and equol (>50 M) dramatically decreased the viability of MCF-7 cells. In addition, the induction of apoptosis was enhanced by the combination of equol (100 μM) and 4-OHT (10 μM) more potently than each compound alone, suggesting that equol increases the effectiveness of tamoxifen. Together, these findings lend credence to the hypothesis that equol and tamoxifen activate the intrinsic apoptotic pathway more effectively than each drug does on its own [144].

Together with adenoviral mutations, the compounds curcumin, genistein, epigallocatechin-gallate, equol, and resveratrol effectively killed both androgen receptor-positive (22Rv1) and -negative (PC-3, DU145) cell lines. The combination of the wild-type virus (Ad5) and AdΔΔ with the antioxidants equol and resveratrol was used to demonstrate synergistic cell death [145]. All three combination-treated cell lines showed a three- to eight-fold reduction in the EC_50_ values for viruses and phytochemicals, respectively. When combined with wild-type virus or AdΔΔ, equol and resveratrol significantly increased the level of apoptotic cell death in PC-3 and DU145 cells, despite the fact that they alone only slightly increased apoptosis in PC-3 and DU145 cells. AdΔΔ and equol or resveratrol were used in in vivo experiments at suboptimal levels, which resulted in less tumor growth without adverse effects on normal tissue. Overall, this study showed that AdΔΔ, an oncolytic adenoviral mutant that increases drug-induced apoptosis in cells, can also interact synergistically with dietary phytochemicals such as equol and resveratrol [145].

## 7. Role of Nanotechnology and Clinical Studies Using Equol

While the spectrum of therapeutic actions of phytochemicals continues to grow (Sak, 2022), it is essential to address certain drawbacks to optimum efficacy. These primarily include low bioavailability and a short residence time in the intestine due to extensive metabolism and instability of these phytoconstituents, thereby limiting their applications [146]. In order to combat these disadvantages, nanotechnological approaches may be applied to improve the solubility of these natural compounds, thereby increasing their bioavailability. Nanoparticles are a commonly used approach in drug delivery, and are widely used in cancer therapy. Studies conducted to analyze the effect of flavonoids with other synthetic anticancer drugs and compounds of natural origin have indicated promising results, thereby indicating more widespread applications in clinical settings [147]. 

As discussed, the spectrum of action of phytoconstituents and products of their metabolism, such as equol, remain limited. In addition to this, due to the contrasts in the pharmacokinetic profiles of different agents, their movement across barriers such as the blood–brain barrier (BBB) and intestinal barrier is variable [148]. This may be enhanced by implementing strategies such as loading onto nanostructured polymers, complexation, and nanostructured lipid carriers, thereby improving the stability and delivery of these agents to the target site [149]. In order to improve the aqueous solubility and thereby oral bioavailability of these agents, strategies such as the usage of carrier complexes and co-crystallization may be employed. This may be used to counter degradation and extensive metabolism for the improvement of the dissolution of these compounds, as well as facilitating target-specific delivery. 

The antioxidant potential of polyphenols continues to be at the forefront of research, owing to their role in the amelioration of a wide variety of metabolic disorders. Keeping pace with the growing importance of environmentally friendly synthesis and drug delivery, green nanoparticles may be employed in therapy. They offer advantages such as uniformity in structure, as well as effective scavenging of reactive oxygen species (ROS) [150]. Nanotechnology may be employed in the delivery of phytoconstituents to overcome multi-drug resistance (MDR), utilizing the principle of controlled release in the management of pathogenic infections. Some of the strategies include the formulation of liposomes (for the delivery of smaller molecules and those with different solubility profiles), hydrogels (for drug entrapment and regulation of drug release), polymeric nanomicelles (for the lowering of toxicity and improved drug bioavailability), and nanofibers (improved biocompatibility and easy modifications of the solubility profile) [151]. While the therapeutic potentials of equol continue to be explored, greater advancements in nanotechnological delivery are required. An increased focus on the designing of nanoformulations, as well as undertaking pre-clinical and clinical studies, would help to further improve our understanding of this molecule, as well as to investigate newer avenues of application. Table 3 represents the current status of clinical studies using equol as a therapeutic molecule. 

## 8. Conclusions and Future Perspectives

In this review article, different types of bioactivities of the degradation product of soy isoflavone daidzein, i.e., equol, are described in different types of malignant models. These bioactivities comprise the effects of equol on hormonal balance by binding to estrogen receptors, but its has also showed anti-inflammatory-, antiproliferative-, antimetastatic-, and apoptosis-inducing effects. All the results described in this review show the high potential of equol in the fight against cancerous neoplasms. Looking to the future, further studies should identify the bacterial species in the colon, which are involved in the biosynthesis of equol from its precursor, daidzein, thereby allowing to understand the mechanisms more thoroughly behind the bacterial production of equol and, if possible, including these species in some food products containing soy isoflavones. In addition, structure-activity analyses might be useful for further improving the anticancer properties of equol to make this molecule semi-synthetically even more potent for the use against tumors in the future.

## Figures and Tables

**Table 1:** Anticancer effects of daidzein and its metabolites including equol based on in vitro studies.

Type of Cancer	Cell Lines	Effects	Mechanisms	Concentration	References
Osteosarcoma	143B and U2OS	Induces apoptosis	↓ proliferation and migration of 143B and U2OS osteosarcoma cells, ↑ %age of S phase cells, ↓ %age of G0/G1 phase cells, ↓ p-Src-ERK, ↓ p-Src, ↓ p-ERK, No change in expression levels of Src, JNK, p-JNK, ERK, p38 and p-p38	Daidzein—0, 10, 20, 50, 100, 200 or 500 µM	[15]
	MG-63	Induces apoptosis	↑ ROS, ↓ mitochondrial membrane potential, ↑ apoptosis rate, ↑ cell cycle arrest at the G_2_/M phase, ↓ Bcl-2, ↓ Bcl-x and ↓ Baid proteins, ↑ Bim protein	Daidzein—IC_50_ value of 59.7 µM	[16]
Colon	HT-29	Induces apoptosis	↓ growth of cancer cells, significant increase in cells in the G_0_/G_1_ phase, ↓ Lipid droplets accumulation, ↓ Perilipin-1, ↓ ADRP and↓ Tip-47 family proteins, ↓ vimentin, ↑ PPAR, ↑ Fas, ↑ FABP, ↑ GPAT3, ↑MTTP, ↓ UCP2. ↓ PI3K, ↑ FOXO3a, ↑ caspase-8	Genistein and Daidzein—0, 25, 50, 100, 200, and 400 μM	[17]
	DLD1, HCT15, COLO205, LOVO, SW480	Induces apoptosis	↓ growth of HCT-15 cells with the expression of ERα and ERβ, ↓ growth of LOVO, and SW480 cells with the ERβ expression, ↑ ERα and ERβ in HCT-15. ↑ ERα and ERβ, ↑ Nrf2	Equol—0, 0.5, 1, 5, 10 μM	[18]
Breast	MCF-7	Induces apoptosis	↑ % age of apoptotic cells, ↑ Caspase 3/7 activity, ↑ Bax, ↓ Bcl2, ↑ ROS, ↓ ERα, ↑ ERβ	Daidzein—IC_50_—50 µM	[19]
	MCF-7 and T47D	Induces apoptosis	↑ cytotoxic effects towards cancer cells, ↓ NGB, ↑p- AKT, ↑ p38 phosphorylation, ↑ cleaved PARP-1	Daidzein—1–10 µM and Equol 1 μM	[20]
	MCF7 and MCF7/ADR	Enhances the anticancer effect of topotecan (tpt) and reverses *BCRP*-mediated drug resistance	↑ anti-proliferative effect with TPT on MCF7 and MCF7/ADR cells, ↑ inhibitory effect of TPT on Topo Ⅰ activity, ↑ inhibition of TPT on the catalytic activity of Topo Ⅰ, ↑ cells arresting at the G_2_/M phase, ↑ apoptosis rate, ↓ resistance of MCF7/ADR cells to TPT, ↓ ERα and BCRP, ↑ TPT accumulation intracellularly	Daidzein—0, 2.13, 6.25, 12.5, 25, 50, 100, 200 and 400 µM and Topotecan 0, 0.78, 1.56, 3.13, 6.25, 12.5, 25, 50 and 100 µM	[21]
	MCF10DCIS.com	Induces apoptosis	↓ TNF-α induced cell migration and invasion, no effect on IκBα expression and NF-κB p65 phosphorylation, ↓ Gli1, ↓ MMP-9	Daidzein—0, 5, 10, 30 and 50 µM and Equol—10 μM	[22]
	MCF-7	Induces apoptosis	↓ MCF-7 viability, ↑ %age of apoptotic cells, ↑ % age cells sub-G_1_ phase, ↓ % age cells in G_0_/G_1_, S and G_2_/M phase, ↑ p53, ↑ p21, ↑ PARP cleavage, ↑ α-fodrin proteolysis, ↑ pro-caspase-7 and pro-caspase-9 cleavage, ↓ Bcl-2, ↑ cytochrome-c release to the cytosol, ↓ Bcl-2: Bax ratio, ↑ tamoxifen’s anti-tumor activity	Equol—0, 25, 50 and 100 μM and 4-OHT 0, 0.01, 0.1, 1.0, 10.0 μM	[23]
	MCF-7 and MDA-MB-453	Induces apoptosis	↓ cell proliferation of cancer cells, ↑ cell cycle arrest in the G_1_ and G_2_/M phases, ↑ %age cells in sub-G_0_ phase, ↓ cyclin D, ↓ CDK2, ↓ CDK4, No Change in the expression of CDK6 and cyclin E, ↓ CDK 1, ↑ p21Cip1 and ↑ p57Kip2, No change in p27Kip1	Daidzein—1–100 μM	[24]
	MCF-7/MDA MB-231	Induces apoptosis	↓ viability of MCF-7 and MDA MB-231 cell lines, no significant growth inhibition was observed in MCF-10A cells, ↑ no of rounded cells due to shrinkage and condensation of cytoplasm, ↑ apoptotic cells, ↑ tunnel +live cells, ↑ ROS, ↓ ∆ψm, ↓ Bcl-xL, ↑ BAX, ↑ Caspase 3/7/9, ↑ cleaved PARP, ↓ PI3K, ↓ p-Akt, ↓ p-mTOR, ↑ affectivity of Centchroman	Centchroman—1–30 µM and Daidzein 10–200 µM	[25]
	MCF-7 and MDA-MB-231	Induces apoptosis	↑ MRP2, ↓ MRP1, ↓ ABCC2 and ABCC1 mRNA	Daidzein—0.05, 0.5 and 5 µM, R-equol and S-equol—0.1, 1 and 10 µM	[26]
	MCF-7	Enhances apoptosis-inducing activity of genistein	↑ cytotoxicity of genistein, ↑ G_2_/M phase cells, ↓ G_1_/S blockade and G_2_/M progression ↑ sub-G_0_/G_1_ population ↑ apoptosis rate, ↑ Bax/Bcl-xL expression ratio, No change in activities of Akt and mTOR, ↑ c-PARP	Genistein—0–100 µM, Equol—0–100 µM	[27]
	MDA-MB-435 (ER)	Induces apoptosis	↑ eIF4GI, ↑ c-Myc, ↑ Cyclin D ↑Bcl-XL ↑ p120 catenin	(R, S) Equol—25 μM	[28]
	MDA-MB-231	Inhibit metastasis	↓ invasive capacity, ↓ MMP-2, No Change on n the expression levels of MMP- 9, TIMP-1 or TIMP-2	Daidzein, R—and S-Equol—0, 2.5, 10, 50 µM	[29]
	MCF-7	Induces apoptosis	↑ ROS, ↓ Bcl-2, ↑ Bax, ↑ release of cytochrome C from the mitochondria into the cytosol, ↑ caspase-9, ↑ caspase-7	Daidzein—25–100 µM	[30]
	MCF-7	--	↑ antiproliferative effects, ↑ pS2 mRNA	Daidzein and (±)-equol 0.001 to 50 µM	[31]
Lung	A594 and 95D	Induces apoptosis	↓ proliferation and colony formation property of cancer cells ↓ IL-6, ↓IL-8, ↓ p65-NFκB expression and activation, ↓ level of p65-NFκB upregulation induced by C/EBPβ	Daidzein—0, 5, 10, and 25 μM	[32]
	A549, HepG-2	Induces apoptosis	↑ ROS, ↓ mitochondrial membrane potential, ↑ apoptosis rate, ↑ cell cycle arrest at the G_2_/M phase, ↓ Bcl-2, ↓ Bcl-x and ↓ Baid proteins, ↑ Bim protein	Daidzein—IC_50_ value of 59.7 µM	[33]
Gastric	MGC-803	Induces apoptosis	↓ viability of MGC-803 cells, ↑ G_0_/G_1_ cell cycle arrest, ↓ CDK2/4, ↓ Cyclin D1/Cyclin E1 ↑ P21WAF1, ↑ apoptosis frequency, ↑ cleaved PARP, ↑ caspase-3. ↑ P-Akt (Ser473 and Thr308)	Equol—5, 10, 20, 40, or 80 μM	[34]
	BGC-823	Induces apoptosis	↓ growth and proliferation of gastric carcinoma cells, ↓ mitochondrial transmembrane potential ↑ cleaved PARP, ↑ cleaved caspase-9, ↑ cleaved caspase-3, ↑ Bax, ↓ Bcl-2, ↓ Bcl-2/Bax	Daidzein—0, 20, 40, and 80 µM	[35]
Hepatocellular	SMMC-7721 and HepG2	Induces apoptosis	↓ proliferation, migration and invasion of cancer cells, ↓ concentrations of pyruvate, glutamate and glucose, ↓ activities of hexokinase, phosphofructokinase and pyruvate kinase, ↓ pyruvate kinase M2, ↑ levels of glycerophosphocholine, ethanolamine, taurine, fumarate, leucine, acetate, ↓ levels of pyruvate, glutamate, glutamine, adenosine monophosphate, creatine, glycine	(−)—5-hydroxy Equol—0, 10, 20, 30, 40 and 50 µM	[36]
	SMMC-7721	Induces apoptosis	↓ proliferation of SMMC-7721 cells, ↑ apoptosis frequency, ↑ S-phase cell cycle arrest, ↑ p21, ↓ cyclin A2; No change in expression of cyclin D1 and H2AX, ↑ caspase-9, ↑ caspase-3, ↑ c-PARP, ↑ Bax ↓ Bcl-2, ↑ caspase-8, ↑ caspase-12, ↑ Chop, ↑ Bip	(±)—Equol, R-(+)-Equol, and S-(–)-Equol—0, 5, 10, 20, 50, and 100 µM	[37]
	SK-HEP-1	Induces apoptosis	↓ cell proliferation of cancer cells, ↑ Prdx-3, ↑ Bak, ↓ Bcl-2, ↓ Bcl-xL, ↑ release of mitochondrial cytochrome c to cytosol, ↑ APAF-1, ↑ caspase 9, ↑ caspase 3	Daidzein—0, 200, 400 and 600 µM	[38]
Pancreatic	MiaPaCa-2 and PANC-1	Induces apoptosis	↓ growth and proliferation of pancreatic cancer cells, inhibitory effects on both ER positive and negative pancreatic cancer cells	Daidzein—0.1, 1, 10, 25, 50, 75 and 100 µmol/L	[39]
Colorectal	SW620	Anti-proliferative effects	↓ p-ERK/ERK, ↓ p-AKT/AKT	Chrysin IC_50_ values 70 µM and Daidzein IC_50_ values 23.5 µM	[40]
	HCT-15	Induces apoptosis	Racemic equol ↓ proliferation of HCT-15 cells, whereas(S) equol had no effect on the proliferation of HCT-15 cells. Racemic equol ↓ ERβ and ↓ Nrf2, while (R) equol ↓ Nrf2	Racemic equol and equol enantiomers—0, 0. 5, 1, 5 and 10 μM	[41]
Bladder	RT112, RT4 and SW780	Induces apoptosis	↓ cell viability, Impaired colony formation, ↑ G1/S cell cycle arrest, ↑ apoptosis frequency, ↓ FGFR3 signaling pathway, ↓ p-FGFR3, ↓ p-Akt, p-ERK	Daidzein—0, 0.5, 1, 2.5, 5, 7.5, 10, 50 and 100 μM	[42]
Prostate	DU145, LNCaP and PC3	Induces apoptosis	↑ cytotoxic activity, ↑ ERβ binding activity, ↑ ERβ gene expression, ↓ cMYC, ↓ Cyclin D1 genes, ↑ caspase 3 and 9, No change in uterotropic and anti-androgenic activities	Novel daidzein molecules—1, 5, 10, 50, 100, 200, 300, 400, 500 µM	[43]
	LNCaP, DU145 and PC3	Induces apoptosis	↑ cell cycle arrest in the G_2_/M phase↓ Cyclin B1 ↓ CDK1, ↑ p21 and p27, ↑ apoptosis rate, ↑ FasL ↑ Bim. ↑ FOXO3a, ↓ p-FOXO3a, ↑ nuclear stability of FOXO3a, ↓ MDM2	S-Equol—0, 0.5, 1, 5, 10 μM	[44]
	DU145	Induces apoptosis	↓ cell migration and invasion, ↓ MMP-, ↓ u-PA, ↓ secreted MMP-2 and MMP-9, ↑ SOD, ↑ Nrf2, ↑ PTEN	(±) Equol 5, 10, 50 µM, Daidzein and Genistein—0.5, 1 and 5 µM	[45]
	PC3, DU145 cells	Induces apoptosis	↓ MMP-2, ↓ MMP-9, ↑ ERγ, No change in Erβ	Equol—0, 0.5, 1, 5, 10 μM	[46]
Choriocarcinoma	JAR and JEG-3	Induces apoptosis	↓ cell viability, ↑ early and late apoptotic cells, ↑ apoptosis frequency, ↑ caspase-9, ↑ caspase-3, ↑ c-PARP, ↓ Bcl-2/Bax	Daidzein—0, 25, 50 or 100 µM	[29]
Cervix	BEL-7402, HeLa,	Induces apoptosis	↑ ROS, ↓ mitochondrial membrane potential, ↑ apoptosis rate, ↑ cell cycle arrest at the G_2_/M phase, ↓ Bcl-2, ↓ Bcl-x and ↓ Baid proteins, ↑ Bim protein	Daidzein—IC_50_ value of 59.7 µM	[47]
Ovarian	caov-3, OVAcAR-3, SKOV3 and A2780	Induces apoptosis	↑ antiproliferative effects on SKVO3 cells, SKOV3 cancer cells became rounder, shrunken and detached from the substratum, ↑ apoptotic cells, ↑ release of cytochrome c into the cytoplasm, ↑ cytosolic levels of cyt c, ↑ Bax, ↑ cleaved caspase-3 and -9, ↑ cleaved PARP, ↑ G_2_ phase cells leading to G_2_/M cell cycle phase arrest, ↓ pcdc25c (Ser216), ↓ cdc25c, ↓ pcdc2 (Tyr15), ↓ cdc2, ↓ cyclin B1, ↑ p21, ↓ migratory capability of cancer cells, ↓ MMP-9, ↓ MMP-2, ↓ p-MEK, ↓ p-ERK	Daidzein—0, 10, 20 and 40 µM	[48]

**Table 2:** Anticancer effects of daidzein and its metabolites including equol based on in vivo studies.

Type of Cancer	Animal Models	Effects	Mechanisms	Dosage	Duration	References
Osteosarcoma	BALB/c nude mice xenografted with 143B (1 × 10^7^) cells	Inhibited Tumor Growth	↓ volume and weight of the tumors, ↑ number of necrotic cells, no systemic toxicity	Daidzein—20 mg/kg	16 days	[15]
Breast	Athymic nude mice xenografted with MCF7 cells (5 × 10^6^ cells)	Inhibited Tumor Growth	No obvious damage found in visceral organs of MCF7 xenograft nude mice, ↓ tumor volume, ↑ tumor inhibition rate of the combination group, ↑ Bax, ↑ p53 and ↑ p21 in the combination group than in the TPT monotherapy group, ↓ Bcl2	Topotecan—3 mg/ kg and Daidzein—5 mg/kg.	15 days	[21]
	MCF-7 cells implanted in ovariectomized athymic mice	Inhibited Tumor Growth	No significant difference was observed in uterine weight, No significant induction of pS2 mRNA (an estrogen responsive marker) in tumors	Daidzein—125, 250, 500 and 1000 p.p.m and (±)-Equol—250, 500 and 1000 p.p.m	7 days	[31]
Lung	Balb/c nude mice xenografted with A549 cells	Inhibited Tumor Growth	↓ Ki-67 and ↓ p65-NF-κB, ↓ tumor volume	Daidzein—5 mg/kg.	21 days	[32]
Bladder	Nude mice xenografted with RT112 cells (1 × 10^6^ cells)	Inhibited Tumor Growth	↓ tumor volume and weight and size, ↓ tumor volume toxicity of daidzein to normal cells,	Daidzein—10 mg/kg and 20 mg/kg	27 days	[42]
Colorectal	Albino rats subcutaneous injected with DMH (40 mg/kg)	Inhibited Tumor Growth	↓ NO, ↓ MDA, ↑ GSH, ↓ CYP2E1 colon content, ↓ CXCL1, ↓ AREG level, ↓ colon content of MMP-9↓ DMH+DSS induced histopathological changes at both doses	Chrysin—125 and 250 mg/kg and Daidzein—5 and 10 mg/kg	56 days	[40]
Prostate	C57B1/6 male mice xenografted with PC3 cell (5 × 10^4^)	Inhibited Tumor Growth	↑ doxorubicin anti-tumor activity, ↑ number of necrotic cells, shows hyperplastic acini lined by simple columnar epithelium and basal cells, ↑ recovery from prostate cancer	The novel metabolites 1 and 2–30 mg/ kg and Doxorubicin—6 mg/kg	21 days	[43]
	BALB/c nude mice xenografted with PC3 cells	Inhibited Tumor Growth	↓ volume and weight of the tumors, ↑ no. number of necrotic cells, ↓ p-FOXO3a and ↑ nuclear stability of FOXO3a	Daidzein	--	[44]
Ovary	Nude mice xenografted with SKVO3 cells (5 × 10^6^ cells).	Inhibited Tumor Growth	↓ Ki-67 ↑ cleaved caspase-3	Daidzein—10, 20 and 40 µg/kg	27 days	[48]

**Table 3:** Clinical applicability of equol in the management of human health.

Agent Administered, Dosage and Duration	Volunteers	Design	Outcome	Conclusion	Reference
10 mg/day supplement containing 98% equol, for 3 months	57 post-menopausal women	Single center, randomized, controlled clinical trial	Reduction in visceral fat, as well as the levels of LDL and total cholesterol, some indications of delayed skin ageing	Equol supplementation may be used for the management of excess visceral fat, and may be used for the alleviation of climacteric symptoms as well as metabolic disorders	[152]
40 g low-fat milk powder + 63 mg daidzein, for a period of 6 months	270 post-menopausal, equol-producing women	Double-blind, randomized, placebo controlled clinical trial	Lowering of T4 levels following daidzein supplementation, no disruption of thyroid functioning	Daidzein supplementation was found to be safe and did not hamper the levels of key markers of thyroid functioning, thereby establishing its safety profile	[153]
Supplement containing 10 mg equol + 10 mg resveratrol + 150 mg quercetin + 178 mg Passiflora, administered up to 8 months	126 post-menopausal women	Clinical trial	Improved vaginal health index, stabilization of pH, improvement of dyspareunia	Equol supplementation may be used for relieving post-menopausal symptoms	[154]
20 g/day soy isolate supplementation, containing 41 mg of isoflavones	44–75-year-old men predisposed to prostate cancer recurrence, following prostatectomy	Randomized, placebo-controlled clinical trial	Slight improvement in hemoglobin and hematocrit levels, reduction in blood pressure in non-producers of equol	The observation from the trial helped to establish the relationship between the equol-producing status of volunteers and soy supplementation, suggesting that certain effects may be observed in each sub-type that may be different from the other, and may be used to enhance therapeutic outcomes	[155]
50 and 100 mg supplementation of phytoSERM, containing genistein, daidzein and *S*-equol, for 12 weeks	71 peri-menopausal women	Double-blinded, randomized, placebo-controlled clinical trial	Good tolerance of the formulation in volunteers, mild adverse effects	Potentials for the usage of equol in the management of post-menopausal symptoms, as well as mild improvement vasomotor and cognitive functioning	[156]
Oral isoflavone administration (150 mg extract), alone or in conjunction with probiotics or hormonal therapy	60 post-menopausal women	Randomized, controlled clinical trial	Alleviation of urogenital complications, increase in the formation of metabolic intermediates, overall improvement of vaginal health	Isoflavone administration may be used in the management of urogenital symptoms and administration with probiotics may be linked with improvement of the synthesis of products of metabolism of isoflavones, which have been established to have therapeutic benefits	[157]
Oral isoflavone administration (consumption of a soy drink providing a dosage of 10–60 mg/day), over 12 weeks	101 post-menopausal women	Randomized, controlled clinical trial	Reduction in the severity of vasomotor symptoms associated with post-menopausal complications	Soy drink supplementation, containing isoflavones, may be used in the management of vasomotor symptoms and may be provided as a natural therapeutic agent	[158]

## Data Availability

Data sharing not applicable.
